# Epidemiological and Clinical Characteristics of Sporadic Creutzfeldt–Jakob Disease: A Retrospective Study in Eastern China

**DOI:** 10.3389/fneur.2021.700485

**Published:** 2021-10-06

**Authors:** Shuo Feng, Xinjing Zhao, Xueying Zhou, Xiang Ye, Xiaolin Yu, Wei Jiang, Yu Deng, Shengnian Zhou, Lin Ma, Peiyan Shan, Guoyu Zhou

**Affiliations:** ^1^Department of Neurology, Qilu Hospital of Shandong University, Jinan, China; ^2^Department of Geriatric Neurology, Qilu Hospital of Shandong University, Jinan, China; ^3^Department of Physical Medicine and Rehabilitation, Qilu Hospital of Shandong University, Jinan, China; ^4^Department of Environmental Health, School of Public Health, China Medical University, Shenyang, China

**Keywords:** prion disease, sporadic CJD, clinical, survival time, eastern China

## Abstract

**Objective:** We aimed to characterize the epidemiological and clinical characteristics of sporadic Creutzfeldt–Jakob disease (sCJD) in eastern China in this retrospective study.

**Methods:** This study enrolled 67 patients with sCJD hospitalized in a grade-A tertiary hospital in eastern China from January 2010 to January 2020. Demographic data, clinical symptoms, brain magnetic resonance imaging (MRI), electroencephalogram (EEG), cerebrospinal fluid (CSF) 14-3-3 protein test, polymerase chain reaction (PCR), and DNA sequence determination of genes were collected and analyzed.

**Results:** There were 62 patients with probable sCJD and 5 patients with possible sCJD. Male (28 cases) to female (39 cases) ratio was 1:1.39. Mean age at disease onset was 64.42 ± 9.00 years (range: 29–88 years), and mean survival time was 9.39 ± 12.58 months (range: 1–60 months for patients who received the follow-ups). The most common onset symptoms were dementia (49.25%), movement disorder (44.78%), and visual disturbance (22.39%), while the most frequent clinical manifestations were language disorders (74.63%), ataxia (70.15%), and myoclonus (70.15%). The positive rates of brain MRI abnormalities, 14-3-3 protein in CSF, and periodic sharp wave complexes (PSWCs) on EEG were 84.90, 68.00, and 46.03%, respectively. The 14-3-3 protein positive (*p* = 0.033) and PSWCs on EEG (*p* = 0.020) acted as the favorable and unfavorable factor for over 1 year of survival time, respectively.

**Conclusions:** There were some differences in epidemiological and clinical characteristics among patients in China and those of other countries. The prognosis and its influencing factors were relatively unexplored in China. The mean survival time of Chinese patients was longer than that of Caucasian patients but shorter than that of Japanese patients. The 14-3-3 protein in CSF and PSWCs on EEG were both closely related to the survival time. It is necessary to promote autopsy or biopsy to better understand sCJD in China.

## Introduction

Prion diseases (PrDs), also known as transmissible spongiform encephalopathies (TSEs), are a group of rare, rapidly progressive and fatal central nervous system diseases attacking humans and animals. According to the modes of human PrDs, PrDs can be classified into three different categories: sporadic (spontaneous), genetic (familial, inherited), and acquired (infectious, transmitted). The sporadic type, the most prevalent one (85–90%), consists of sporadic Creutzfeldt–Jakob disease (sCJD), rare entities of sporadic fatal insomnia, and variably protease-sensitive prionopathy (VPSPr) (1, 2). sCJD is generally regarded as a spontaneous neurodegenerative illness, arising either from templated misfolding and protein conformation change of PrP^C^ (normal brain prion-related protein) or spontaneous somatic mutation of PRNP (gene encoding the PrP^C^). sCJD is clinically characterized by rapidly progressive dementia with ataxia, myoclonus, or other neurologic symptoms (3, 4) and neuropathologically represented by the presence of aggregates of abnormal prion protein, spongiform change, neuronal loss, and gliosis (5). At least six molecular pathological subtypes are classified by genotypes of PRNP codon 129 and protease cleavage sites of PrP^Sc^ (misfolded disease-causing forms), which includes MM1, MV1, VV1, MM2, MV2, and VV2. They are responsible for the diversity of clinical characteristics like onset age, survival time, and symptoms (6, 7).

sCJD is a globally distributed disease. The identification of disease phenotype spectrum has greatly increased the possibility of early diagnosis of sCJD subtype specifically, which is also the foundation of developing effective treatments. In China, the CJD surveillance program supported by the Chinese Center for Disease Control and Prevention (CCDC) was conducted since 2006. sCJD cases (261) were reported nationwide from 2006 to 2010, with a higher number of cases in the east compared with those in other regions (8). However, the prevalence of sCJD has been rarely reported in the past decade in China. Besides, clinical features such as gender ratio and survival time are not consistent in several Chinese studies. Data on the prognosis of Chinese sCJD patients is even more deficient (8–14), and only one study had analyzed the factors affecting it (11). It is urgent to understand the current situation of sCJD in China. Here we presented a retrospective study on the epidemiologic and clinical characteristics and the prognoses of 67 sCJD patients in eastern China to raise awareness of this rare neurodegenerative disease.

## Materials and Methods

### Patients Enrolled

In this study, 67 suspected sCJD patients were enrolled from January 2010 to January 2020. All patients were admitted to the Department of Neurology, Qilu Hospital of Shandong University, which is a grade-A tertiary hospital in eastern China. Suspected sCJD cases were diagnosed according to the amended diagnostic criteria for CJD updated by Hermann et al. in 2018 (15). In brief, the possible diagnosis of sCJD is based on the clinical manifestations, which are progressive dementia and at least two out of four following signs: myoclonus, visual or cerebellar disturbance, pyramidal or extrapyramidal dysfunction, and akinetic mutism. The probable diagnosis of sCJD needs further clinical data, such as typical changes in EEG and MRI, or laboratory data, such as positive 14-3-3 protein in CSF. An absolutely definite diagnosis of any form of CJD usually requires either pathological or pathogenic examination of brain tissues. All patients had no history of blood transfusion, potential iatrogenic exposure from human cadaveric pituitary hormones, dura-mater implants, corneal grafts or neurosurgery. No patient had a family history of PrDs. Patients with diseases similar to sCJD, such as Hashimoto's encephalopathy, intoxication, Wernicke–Korsakoff syndrome, autoimmune encephalitis, and paraneoplastic syndrome, were excluded. This study was in compliance with the ethical principles of the Declaration of Helsinki and was approved by the ethical committee of the Qilu Hospital of Shandong University (KYLL-202011-189).

### Data Collection

We examined the medical information and clinical records including demographic data, onset time, clinical manifestations, the results of neurologic physical examination, and specific auxiliary examinations. The following data were collected and analyzed: brain MRI scanning, electroencephalogram, cerebrospinal fluid 14-3-3 protein test, PCR and DNA sequence determination of PRNP gene from blood samples. Telephone-based follow-ups were carried out by professional neurological physicians to collect the information of disease outcome and survival time.

The neuropsychiatric symptoms of patients were defined as at least one of the following manifestations: agitation, depression, aggression, apathy, personality changes. Brain MRIs were performed using a 3.0-T superconducting magnet (GE Healthcare, Pittsburgh, PA, USA) and included T1, T2, fluid attenuated inversion recovery (FLAIR), and diffusion-weighted imaging (DWI) sequences. EEGs were performed by at least two technicians with relevant qualifications independently, and all cerebrospinal fluid samples were sent to the CCDC to detect 14-3-3 protein by Western blot. Survival time was calculated with the disease onset time and the death date.

### Statistical Analysis

All data were analyzed using SPSS 26.0 software (IBM Corp., Armonk, NY, USA). Descriptive statistics were performed on the demographic characteristics. Continuous variables were presented in the form of mean ± standard deviation (SD) and compared by Student's *t*-test. Categorical variables were expressed as frequency or percentage, while comparison was done with Chi-square test and Fisher's exact test. Kaplan–Meier survival analysis was conducted for deceased patients. Cox proportional hazards regression model and forest plot were applied to describe and analyze the survival time. Statistical significance was set at *p* < 0.05.

## Results

**Figure 1:**
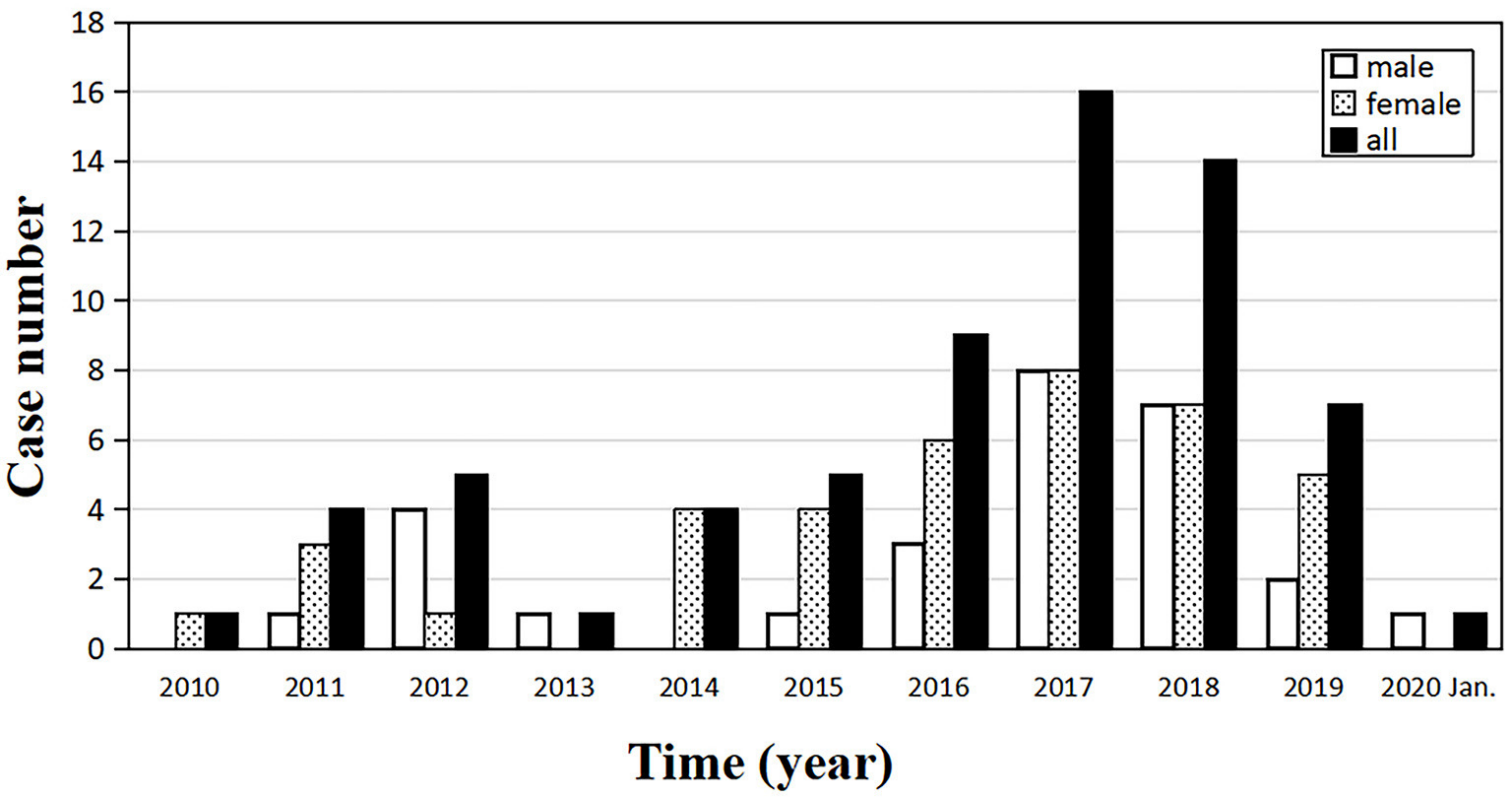
Annual distribution of the number of sporadic Creutzfeldt–Jakob disease (sCJD) patients, from January 2010 to January 2020 (*n* = 67).

In the past 10 years, the number of clinically confirmed sCJD cases in our center is on the rise generally, reaching the highest level of 16 in 2017 (Figure 1). At the end of the study, 43 cases were followed up and 39 cases died.

### General Demographic Features

**Figure 2:**
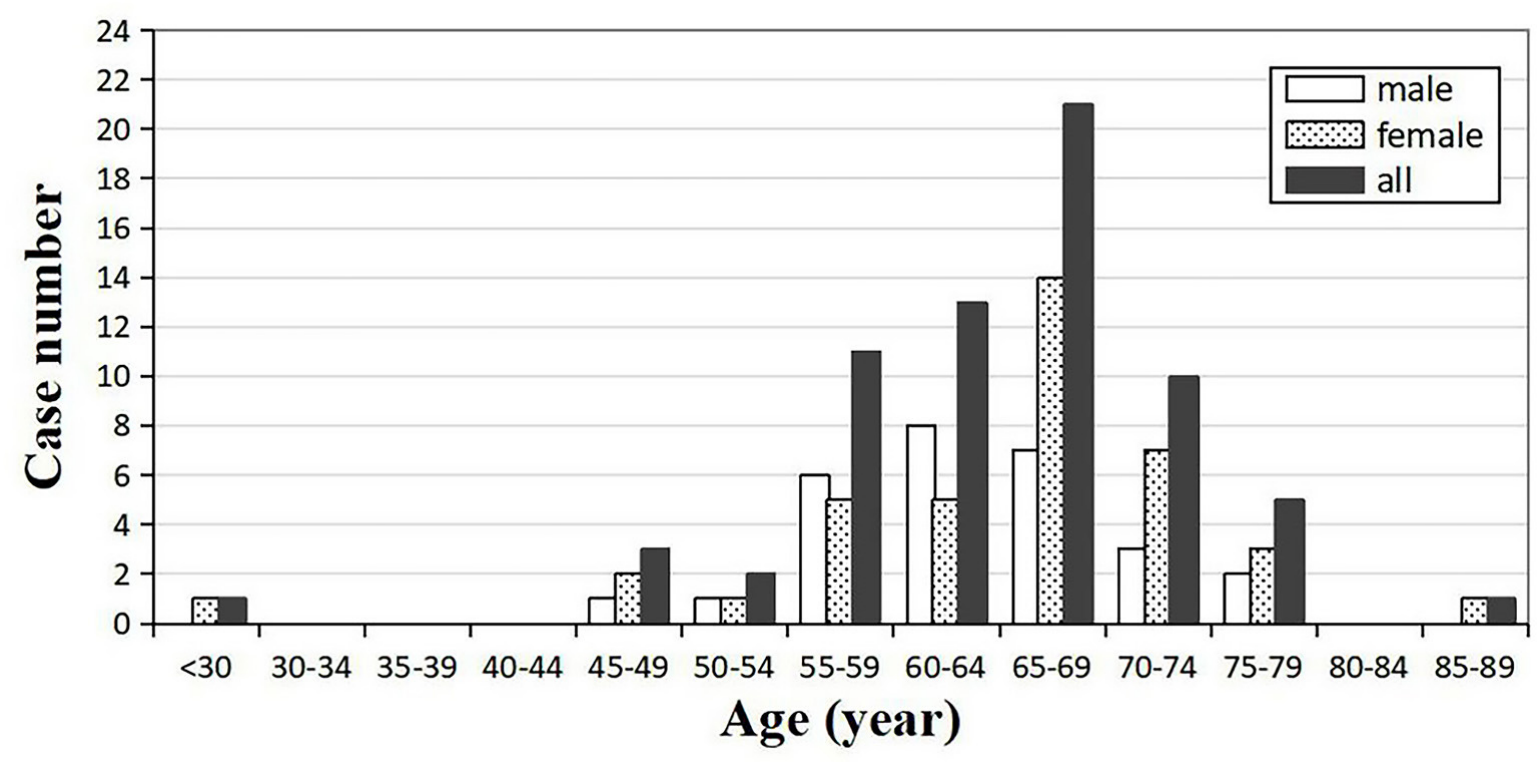
Distribution of the onset age and gender-specific onset age in sCJD patients (*n* = 67).

Out of 67 enrolled cases, 62 were probable diagnosis of sCJD, and 5 cases were possible diagnosis of sCJD. There were 39 females and 28 males, with a gender ratio of 1.39:1. The age of onset ranged from 29 to 88 years (mean ± SD: 64.42 ± 9.00 years, median: 66 years). The peak of age groups was 65–69 years (31.34%) in all patients, and the same pattern could be observed in the female subgroup. For males, the peak was 60–64 years, earlier than females (Figure 2). The mean (±SD) and median onset ages of males was 63.57 ± 6.90 and 64 years, respectively (range: 46–75 years). No significant difference was found in the mean age (*p* = 0.519) and the median age (*p* = 0.212) between the male and female groups (Table 1).

**Table 1:** Onset age and survival time of sporadic Creutzfeldt–Jakob disease (sCJD).

**Gender**	**Onset age (years)**				**Survival time (months)**			
	***n***	**Range**	**Mean ± SD**	**Median**	***n***	**Range**	**Mean ± SD**	**Median**
Total	67	29–88	64.42 ± 9.00	66	39	1–60	9.39 ± 12.58	5
Male	28 (41.8%)	46–75	63.57 ± 6.90	64	15 (38.5%)	2–30	8.29 ± 8.28	6
Female	39 (58.2%)	29–88	65.03 ± 10.30	67	24 (61.5%)	1–60	10.64 ± 14.65	4.5
*p* ^a^	–	–	0.519	0.212	–	–	0.647	0.741

a *Difference in mean onset age and survival time between two genders were analyzed by Student's t-test method. Difference in median onset age and survival time between two genders were analyzed by Brown–Moo test method*.

### Clinical Features

Dementia (49.25%), movement disorder (44.78%), and visual disturbance (22.39%) were the most common onset symptoms in 67 patients. Visual disturbance was more predominant in females than in males (*p* = 0.043) (Table 2). Neuropsychiatric symptoms, dizziness, epilepsy seizure, sleep disorders, and language disorders were also observed at the onset of disease. The most common clinical manifestations were language disorders (74.63%), ataxia (70.15%), and myoclonus (70.15%). Patients with movement disorder in this study all manifested as parkinsonism characterized by bradykinesia (53.73%), and two of them had static tremor simultaneously (2.99%). Moreover, 58.21% of the patients developed neuropsychiatric symptoms, with psychotic symptom (26.87%) as the most common one, followed by apathy (19.40%) and panic (13.42%). Akinetic mutism occurred in 37.31% of the patients (Table 2).

**Table 2:** The main clinical and ancillary test features of sCJD patients.

**Clinical and ancillary test features**	**Total**	**Onset age (years)**			**Gender**		
	***n* = 67**	** <60 (*n* = 17)**	**≥60 (*n* = 50)**	***p* ^**d**^**	**Male (*n* = 28)**	**Female (*n* = 39)**	***p* ^**d**^**
**Onset symptoms**	67						
Dementia	34	9	25	0.834	17	17	0.167
Movement disorder	30	8	22	0.827	13	17	0.818
Visual disturbance	16	5	11	0.536	3	13	0.043^*^
Neuropsychiatric symptoms	6	0	6	0.325	3	3	0.688
**Symptoms following disease progression**	67						
Visual disturbance	25	9	16	0.123	8	17	0.210
Ataxia	47	14	33	0.203	18	29	0.374
Parkinsonism^a^	36	7	29	0.262	12	24	0.100
Pyramidal signs	26	11	15	0.011^*^	10	16	0.660
Language disorders	50	13	37	1.000	21	29	0.445
Akinetic mutism	25	7	18	0.703	13	12	0.069
Myoclonus	47	10	37	0.237	22	25	0.202
Neuropsychiatric symptoms	39	9	30	0.610	18	21	0.393
**MRI abnormalities** ^**b**^	56/66^c^	16	40	0.432	23	33	0.599
Cortex	50	14	36	0.461	29	21	0.902
Basal ganglia	29	9	20	0.385	13	16	0.727
**CSF 14-3-3 protein positive**	17/25^c^	5	12	1.000	9	8	0.673
**EEG PSWCs positive**	29/63^c^	7	22	0.832	7	22	0.006^**^

a *Bradykinesia, static tremor, or both*.

b *Diffusion-weighted imaging brighter than fluid-attenuated inversion recovery*.

c *The numbers after a slash represent the numbers of patients receiving the corresponding ancillary test, while the numbers before a slash represent the numbers of patients with positive results*.

d *Chi square test was used to compare the features of the two groups*.

* *p < 0.05*;

** *p < 0.01*.

*CSF, cerebrospinal fluid; EEG, electroencephalogram; PSWCs, periodic sharp wave complexes*.

### Brain Magnetic Resonance Imaging

Sixty-six patients underwent brain MRI scanning, and 56 (84.85%) of them had typical MRI abnormalities, such as “cortical ribboning,” abnormal signal in basal ganglia on DWI. Among them, 50 cases (75.76%) had “cortical ribboning,” 29 cases (43.94%) showed abnormal signal in basal ganglia (Table 2), and 23 cases (34.85%) had both. Twenty-seven cases (40.91%) had only “cortical ribboning,” while six cases (9.09%) presented abnormalities only in basal ganglia.

### Electroencephalogram

Of the 63 patients who undertook EEG examinations, 29 had PSWCs (positive rate: 46.03%), including 7 males and 22 females. Statistical analyses showed a significant difference in the positive rates of PSWCs between male and female groups, and the female group had a higher positive rate (*P* = 0.006) (Table 2).

### 14-3-3 Protein

A total of 25 patients, including 12 males and 13 females, were tested for cerebrospinal fluid 14-3-3 protein. Seventeen patients were positive (positive rate: 68.00%). The positive rate for males and females was 75.00% (nine positive cases) and 61.54% (eight positive cases), respectively (Table 3). Meanwhile, we managed to complete the follow-ups for 17 of the tested patients, among whom 15 cases were 14-3-3 protein positive. Four patients, whose survival time was more than 24 months, were still alive. We explored the relationship between 14-3-3 protein and other three factors including gender, age, and survival time. Survival time for patients with positive CSF 14-3-3 protein (8.55 ± 6.06 months, median 7 months) was significantly longer than that for patients without 14-3-3 protein (2.00 ± 0 months, median 2 months) (*p* = 0.005) (Table 3).

**Table 3:** The 14-3-3 protein in cerebrospinal fluid (CSF) of sCJD patients.

**14-3-3**	**Gender (*****n*** **=** **25)**				**Onset age (years**, ***n*** **=** **25)**		**Survival time (months**, ***n*** **=** **13)**		
	***n***	**Male**	**Female**	***p* ^**b**^**	**Mean ± SD**	**Median**	***n***	**Mean ± SD**	**Median**
Positive	17	9	8	0.896	65.29 ± 7.05	67	11	8.55 ± 6.06	7
Negative	8	3	5	–	65.63 ± 10.32	67.5	2	2.00 ± 0	2
*p* ^a^	–	–	–	–	0.926	–	–	0.005^*^	–

a *Differences in mean age and mean survival time between two groups (14-3-3 positive and 14-3-3 negative) were analyzed by Mann–Whitney U-test method*.

b *The relationship between 14-3-3 protein positive and gender was analyzed by Spearman correlation analysis method*.

* *p < 0.01*.

### PRNP Gene

PCR and DNA sequence determination of PRNP gene were performed in 25 sCJD patients (12 males and 13 females) in our study. No PRNP gene mutation was found in these 25 cases and 24 of them had the methionine homozygous genotype at codon 129 (MM, 96.00%). Only one was heterozygous for methionine/valine (MV, 4.00%).

### Survival Time

**Figure 3:**
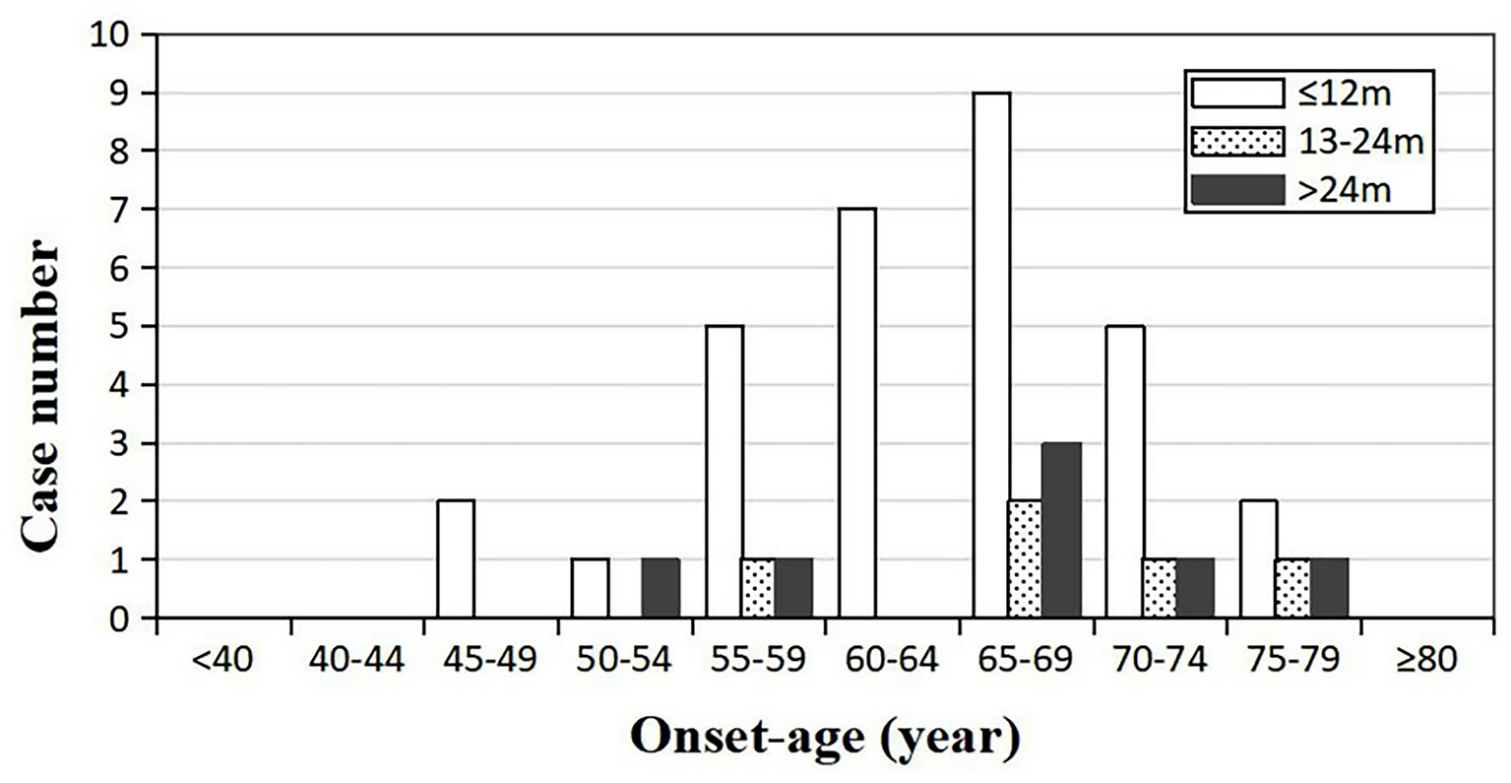
Distribution of the survival time among sCJD patients stratified by onset age (*n* = 43). Across all age groups, patients whose survival times were <1 year tend to be the most, indicating that the survival time may not be necessarily related to the onset age.

**Figure 4:**
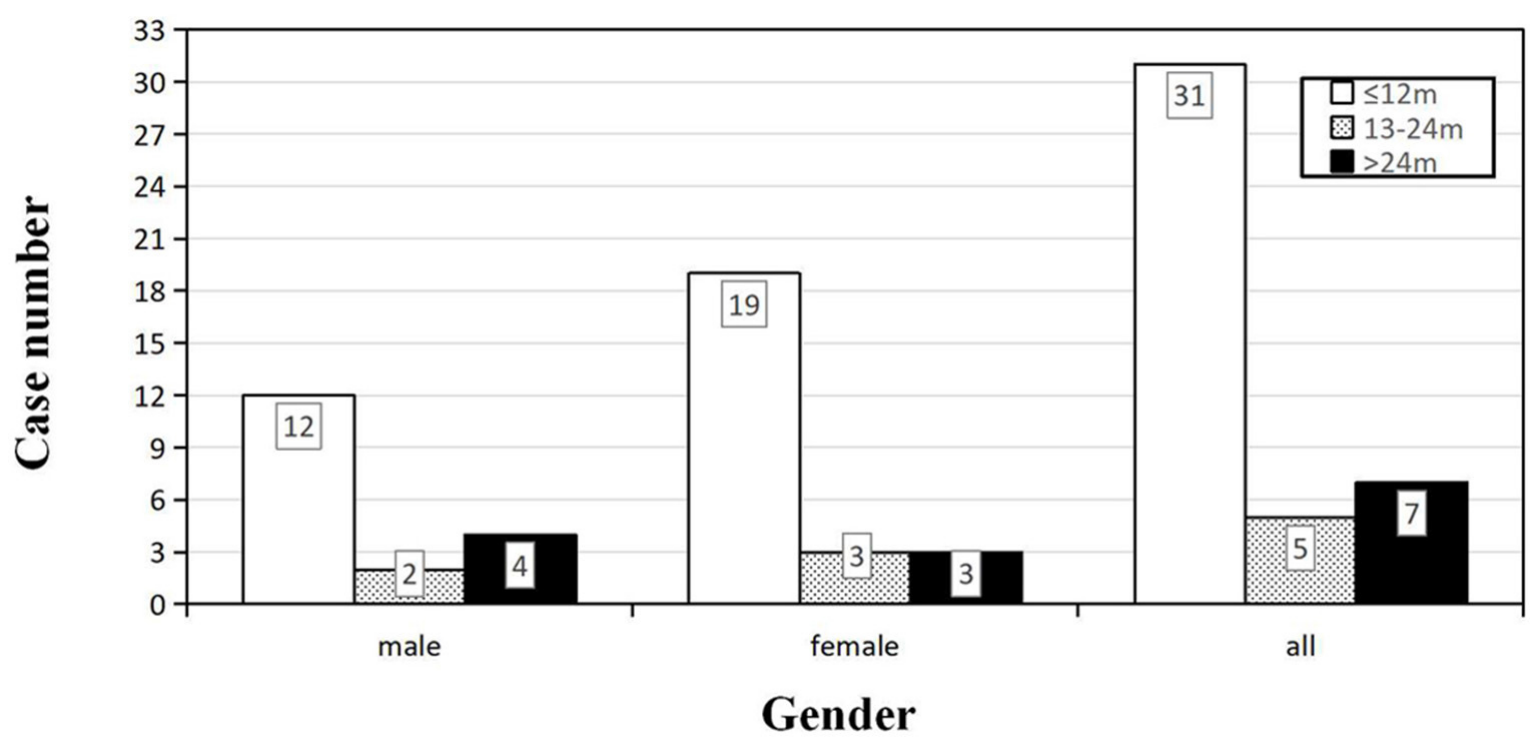
Distribution of the survival time among sCJD patients stratified by gender (*n* = 43). For both male and female, the largest proportion of patients had a survival time within 1 year, indicating that the survival time may not be necessarily related to the gender.

Forty-three patients (19 males and 24 females) received the follow-ups, and 39 patients died. The onset age of the 43 patients was from 47 to 78 years. In each subgroup based on age, most patients lived <12 months (Figure 3). Thirty-one cases (12 males and 19 females) died within 12 months (72.09%), five cases (2 males and 3 females) had a survival time between 13 and 24 months (11.63%), and seven cases (4 males and 3 females) were still alive 24 months after the onset (16.28%) (Figure 4). The mean survival time was 9.39 ± 12.58 months, and the median was 5 months (range: 1–60 months) (Table 1).

**Figure 5:**
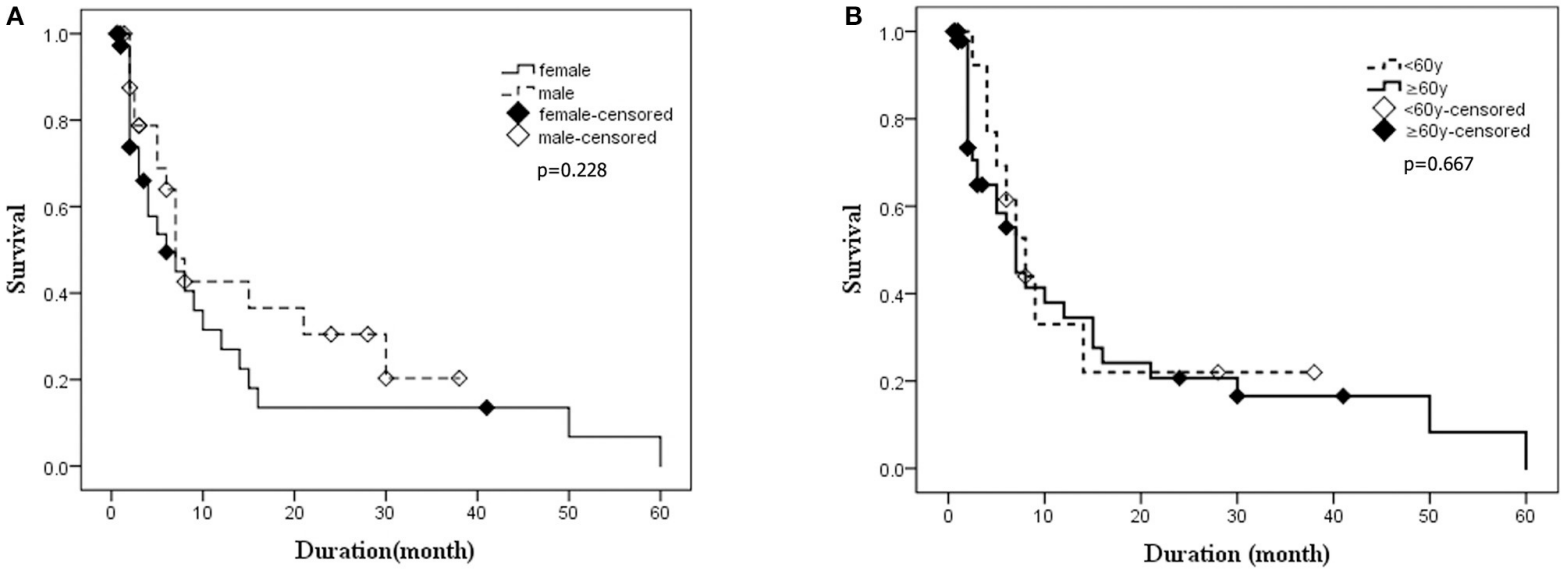
The Kaplan–Meier survival curves for deceased patients. The X-axis represents survival time (months), and the Y-axis represents survival probability. **(A)** Survival time for patients stratified by gender. **(B)** Survival time for patients stratified by age. Graphic symbol shows the median survival time (50% percentile) of the distinct gender or age groups.

**Figure 6:**
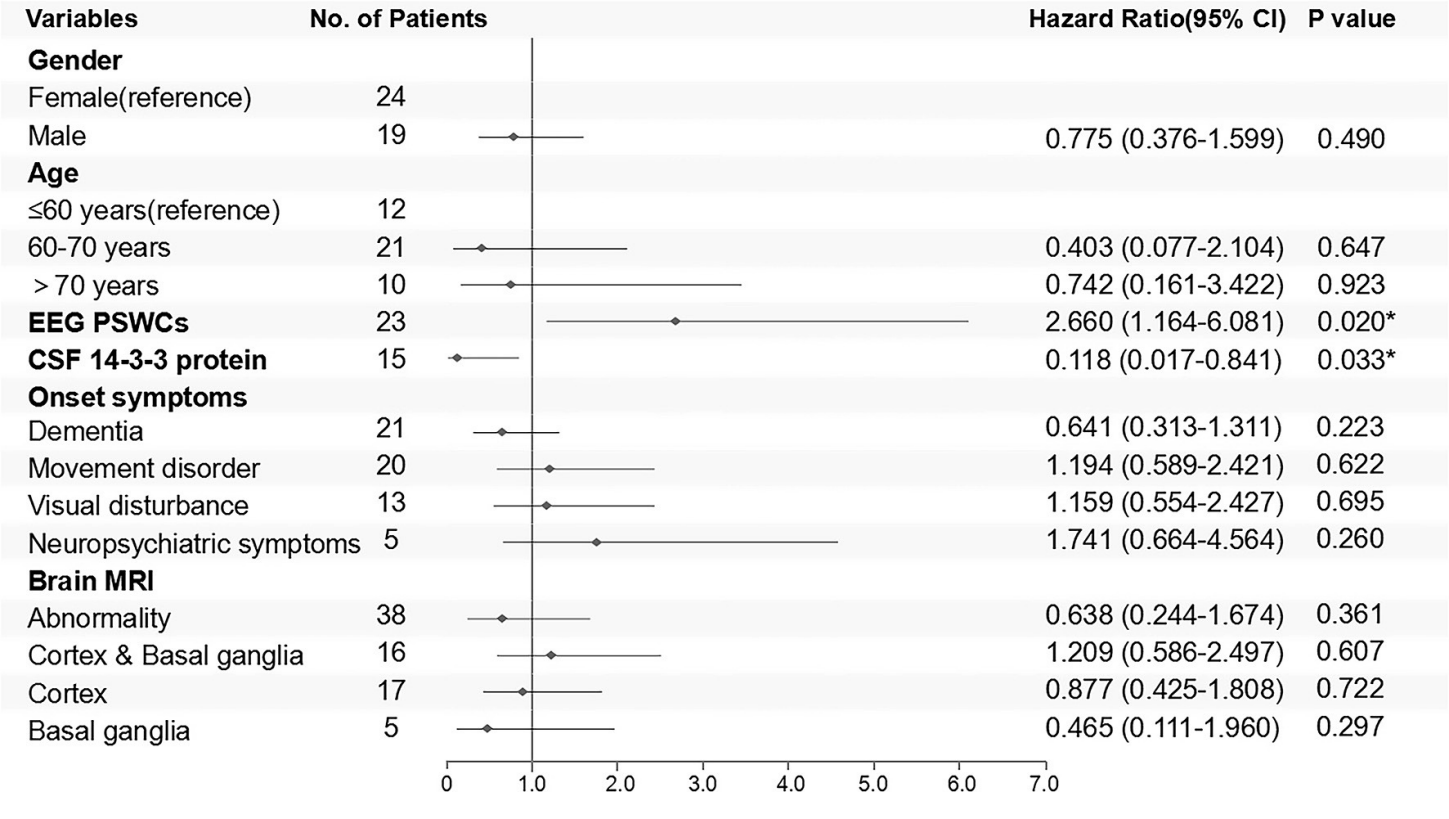
Cox regression model for follow-up patients is shown in the form of a forest plot. Outcomes were classified into survival or death 1 year after disease onset. Negative results of ancillary test were taken as reference.

Kaplan–Meier survival curves for the patients who underwent the follow-ups are shown in Figure 5A (subgroups by gender) and Figure 5B (subgroups by age). No statistical difference of survival time was observed between the groups of different genders (*p* = 0.228) (Figure 5A). The median survival time of patients under 60 years old was 7 months, while that of patients over 60 years old was 8 months. The difference in survival time was not statistically significant between different age groups (*p* = 0.667), either (Figure 5B). Cox proportional hazards regression model was used to analyze factors influencing the survival time. The results showed that 14-3-3 protein in CSF was favorable to over 1 year of survival time, while the PSWCs on EEG was an unfavorable factor for surviving over 1 year after onset (Figure 6).

## Discussion

In the current study, we retrospectively analyzed the demographic and clinical features of 67 sCJD patients in eastern China, and 43 of them received follow-ups. By the end of the study, 39 follow-up patients had died. Generally speaking, though PrDs are considered rare, the number of sCJD cases diagnosed in our hospital has been on the rise over the past 10 years. This may be attributed to the aging population, increasing number of patients admitted to hospital, and improved clinician awareness of the disease. However, the number of cases peaked in 2017 and has since declined mildly. It is for sure that the total number of inpatients from the department of neurology in our hospital has been increasing from 2010 to 2019. Given that the number of CJD cases nationwide in the recent 5 years has not been published, and other Chinese single-center study have not paid attention to the trend of the number of the patients, whether this trend is consistent with the national situation remains unknown.

In this study, we found that there were more female than male sCJD patients, and the female-to-male ratio was 1.39. Two studies of Chinese patients were consistent with our findings, in which the number of females predominated (10, 12), but two other studies had reported a higher proportion of males, where the female-to-male ratio was 0.82 and 0.79, respectively (8, 14). Because of small sample size and various nationalities in China, there might be biases in those results. A convincing gender ratio of sCJD patients in China requires demographic data of multicenter or population-based studies in the future. According to Japanese national sCJD surveillance data from 2001 to 2010, the ratio of female to male was 1.15 (16). In a research with 2,451 pathologically confirmed sCJD patients enrolled by the European Creutzfeldt–Jakob Disease Network (EUROCJD), which covered several European countries, Canada, and Australia, the ratio of female to male was 1.18 (7). In contrast, the data of 116 sCJD cases from the United States demonstrated that the female-to-male ratio was 0.49 (17). It is worth noting that this American study included patients of multiple races including Whites, Blacks, and American Indians. Race, population structure, molecular pathological subtype pattern, and the gender difference in economic status could partly contribute to the disparate ratios by countries. Further researches are required to investigate the exact reasons by controlling multiple factors. What is more, adequate sample size is indispensable in that the patient numbers of Chinese studies are much less than that in other countries.

The mean age of disease onset in this study (64.42 years old) was comparable with the findings from Korea (65.5 years) (18), Japan (65.5 years) (19), the United States (66.15 years), (20) and Europe (66 years) (21), but higher than that of a few Chinese patient groups [58 years (14) 60.3 years (9)]. Another two surveys in China have produced results essentially in agreement with ours at the median onset age, which was 64 years in our study (8, 11). According to these reported Chinese studies, most of suspected patients were not tested for PRNP gene to rule out genetic Creutzfeldt–Jakob disease (8, 9, 11, 14), which might affect the results and analysis of the study. Similarly, there were two patients younger than 30 years old in our study. Although these two patients met the criteria for clinical diagnosis of sCJD, they were not eventually diagnosed by pathological examination, which may also have an impact on the result. At the same time, considering that the different regional demographic features might play a role in the heterogeneity of onset ages among domestic studies, more accurate onset age remains to be verified by large national surveys. Additionally, Kotkowski et al. found that age (66 ± 11.5 years) was correlated with sCJD onset (20). As with many other neurodegenerative diseases, sCJD generally occurs in late adulthood.

So far, no effective treatment has been developed for sCJD, which remains uniformly fatal. In our study, the mean survival time was 9.39 months, and 72.09% of the sCJD patients had a survival time <1 year. Similarly, other researches in China showed that mean survival time ranged from 6.1 to 11.6 months, and 1-year mortality was 68.50–74.00% (11, 13, 14). We also found that 14-3-3 protein and PSWCs acted as the favorable and unfavorable factor for over 1 year of survival time, respectively. The same predictive effect of PSWCs was also observed in two European large-scale studies, in which over 2,000 pathologically definite sCJD cases were incorporated (7, 22). However, some studies have found that 14-3-3 protein was correlated with a shorter survival time (7, 21–24). The possible reasons for this discrepancy may be as follows: first of all, there are only two 14-3-3 protein-positive patients in this study, whose survival times were <1 year. Considering the small sample size and related uncertainties, our findings need to be further validated by large-scale studies. Second, the survival time is usually not determined by a single factor, which is why Llorens et al. have set up a multifactor predictive model (21). To our knowledge, previous study objects were Caucasians, but no Chinese researchers have explored the relationship between CSF biomarkers and survival time. Different races, regional environments, molecular subtypes and some relevant unknown factors may partly explain our different findings. Last but not the least, pathological examinations were not carried out in our patients, which could reduce the accuracy of diagnoses and then affect the results. Pocchiari et al. concluded that there was correlation between survival time and codon 129 type (22). The multinational study carried out by EUROCJD, whose research objects were mainly Caucasians, revealed that the mean survival time was only 5 months, and 85.8% of the cases died within 1 year. The MM, MV, and VV type of codon 129 accounted for 66.1, 17.0, and 16.9%, respectively (7). In Asia, almost all the sCJD patients were MM type (8, 9, 11, 14, 25–27). MM type accounted for 97.0–100.0% of all sCJD patients according to previous Chinese studies, while others were all MV type (8, 9, 11, 14, 27), which was in agreement with our findings. However, due to the extreme paucity of pathological examination, PrP^Sc^ types of Chinese sCJD patients are unavailable till now. As a consequence, different distribution patterns of PRNP codon 129 types may be one of the reasons for the variations in survival time, and other possible reasons remain to be explored.

However, according to a Japanese report, the mean survival time and 1-year mortality was 15.7 months and 48.1%, respectively (26). By comparison, survival time of the patients is shorter in China, where the MM type is the majority in Japan, though (8, 11, 14, 26). It should be noted that there is a proportion of Japanese patients with MM2 type, and nearly all of those survival time was more than 1 year, and some even reached 6 years (28). Additionally, the MM2 type can be further divided into MM2C and MM2T subtypes based on distinctive cortical and thalamic histopathology (2, 29), and the MM2T subtype is also known as sporadic familial insomnia (sFI), which is relatively rare. Their mean survival time was comparable despite different races (28, 30). Studies also have shown that MM1 type patients had an obviously shorter media survival time of 4.0–9.0 months (7, 31). However, the pathological classification of MM type is still not clear in China, which might be the main reason for the different survival time between Japanese and Chinese patients. More pathological results are needed to verify it. What is more, Iwasaki et al. found that tube feeding was an independent factor influencing total disease duration for MM1 type of sCJD (31), which also partly accounted for the longer survival time of Japanese patients (32). Future studies should be conducted by controlling possible interfering factors, such as regions, economic conditions, races, or molecular pathological subtypes, to find the exact factors that affect the course of disease.

The most common initial symptoms in our study were dementia, movement disorder, and visual disturbance, which were consistent with the results of other two centers in China (9, 11). It was reported by a Japanese research that 55.10% of cases started from psychiatric symptoms such as dementia or sleeping difficulties (26). According to a German study of 492 sCJD patients, dementia was the most common initial symptom followed by cerebellar, visual, and psychiatric disturbances (33). An American study with 114 sCJD patients showed that cognitive symptoms, especially memory loss, were the most common manifestations (34). Despite the discrepancies in race, region and even molecular pathological types, dementia is the most frequent onset symptom in these patients. Most notably, 49.25% of our patients rapidly developed progressive dementia, almost all of whom had this symptom at the time of seeking medical treatments.

The diagnosis of sCJD is challenging. Only autopsy can make a definite diagnosis, but most diagnoses of sCJD are based on clinical manifestations and ancillary tests without specific biological markers. Biomarkers with high specificity and sensitivity are urgently needed to assist clinicians in diagnosing sCJD. The first biomarker identified for diagnosis was PSWCs on EEG, which was performed in 63 patients in our study. Only 29 (46.03%) of them showed positive results, but the sensitivity of PSWCs among the confirmed CJD patients was 55.7–64% (7, 35). Since PSWCs usually occur in the late stages of the disease, the timing of test can also affect the positive rates. Serial EEG recordings are required for higher positive rates (36–38). Furthermore, given that PSWCs are common in encephalopathies, metabolic disorders, dementias, and other diseases, the specificity is relatively low (35, 39–41). Another widely used biomarker was 14-3-3 protein in CSF. As a moderately sensitive biomarker (42), 14-3-3 protein showed a positive rate of 68.00% in our cases, not very prominent detecting efficiency. Compared with PSWCs and 14-3-3 protein, brain diffusion-weighted MRI has a higher diagnostic utility in sCJD, with a sensitivity ranging from 92 to 96% and a specificity of about 93 to 94% (43–45). Further studies indicated that specific MRI abnormalities could serve as useful parameters for predicting the clinical course, such as those who have cortical plus basal ganglia hyperintensity on DWI tend to show symptoms of advanced stage (46, 47). The abnormal rate of brain MRI of our patients was 84.85%, which was a bit lower than that of studies with some defined patients (10, 42–44). One of the possible reasons is that our clinical diagnosis of sCJD is not conclusive without pathological inspections. In recent years, some of the newer diagnostic markers have also been explored; total (t)-tau protein in CSF was found to be superior to 14-3-3 protein and neurofilament light chain protein (NfL) as a biomarker with the highest sensitivity of 88.2–90.20% (48) and specificity of 74.5–78.9% (49, 50). The ratio of t-tau and phosphorylated-tau may be useful to distinguish patients with CJD from patients with other dementias (51, 52). In addition, a new PrP^Sc^ amplification assay, named real-time quaking-induced conversion (RT-QuIC), has also shown an overall diagnostic sensitivity of 82.1–92% and a specificity of 98.5–100% (49, 53–56), sometimes behind t-tau. Unfortunately, none of our patients had received the above two tests on account of technical limitations and examination costs. These problems could change with the deepening understanding and research of the disease.

Compared with nearly 100% in Austria (57), 68.7% in the United States (58), 66% in Germany (59), 60% in Australia (60), and 51% in Belgium (61), the autopsy rate is extremely low among Chinese patients due to the influence of traditional Chinese values. Besides, the fear and limited understanding of prion disease, as well as the restriction on the conditions of medical institutions, also affect autopsies on patients with this contagious neurodegenerative disease in China. Biopsy could not be performed smoothly and generally in China, either, which was confirmed by previous domestic literature (8, 10–13). Thus, their vast majority of molecular pathological subtypes remained unknown. To have a more accurate understanding of sCJD in China, the application of autopsy or biopsy in suspected patients is urgently needed.

This study described and analyzed the epidemiological and clinical characteristics of sCJD patients in eastern China systematically. Compared with most previous studies in China, we have gotten a relatively detailed clinical characteristics and complete follow-up data, and conducted a thorough analysis of the relationship between the two. Moreover, in the absence of autopsy and biopsy, compared with to the only study that performed prognostic analysis (11), the newer clinical diagnostic criteria were applied here. Differences in gender, 14-3-3 protein, PSWCs, and contributing factors on survival time were revealed in Chinese patients for the first time. There are also several limitations in our study. First, the number of sCJD cases was relatively small, and CSF 14-3-3 protein and PRNP gene analysis were not performed in all cases. With the ongoing deep research work, the rate of CSF and gene testing for patients with prion disease would increase gradually, thus, raising the accuracy of sCJD diagnosis. Second, some patients were lost to follow-up, resulting in unknown prognoses. In future research, we will expand the sample size and conduct more intensive and longer-term follow-up observations. Third, although the primary diagnostic methods for sCJD are t-tau and RT-QuIC, rather than the 14-3-3 protein and PSWCs now, the first two were not performed in this study. Finally, pathological examination of brain tissue, which is crucial to diagnosis, was not carried out. Along with the further understanding of prion disease and the development of medical and economic conditions in China, this situation is expected to be improved.

## Conclusions

In conclusion, the epidemiological and clinical characteristics in Chinese studies are inconsistent, and there are some differences when compared with other countries. The mean survival time of Chinese patients was longer than that of Caucasian patients but shorter than that of Japanese patients. This study found that 14-3-3 protein in CSF and PSWCs on EEG acted as the favorable and unfavorable factor for over 1 year of survival time of Chinese patients, respectively, which has not been reported in previous literature of China. With an extremely low autopsy rate of prion disease in China, it is necessary to promote autopsy or biopsy to better understand the incidence, clinical characteristics, and the effective factors for prognosis of sCJD or prion diseases. Systematic studies on genes, protein, pathology, and clinical phenotypes are needed for a deeper insight of this disease as well as other possible neurodegenerative diseases related to protein misfolding.

## Data Availability Statement

The raw data supporting the conclusions of this article will be made available by the authors, without undue reservation.

## Ethics Statement

The studies involving human participants were reviewed and approved by the Research Ethics Commission of Qilu Hospital of Shandong University, Jinan, China (KYLL-202011-189). Informed consent was waived by the Research Ethics Commission.

## Author Contributions

GZ designed the study and reviewed the manuscript. GZ, XZha, XYe, XYu, XZho, WJ, SF, SZ, PS, and LM collected the data. GZ, SF, and YD performed the statistical analysis. SF summarized the data and drafted the manuscript. All authors were responsible for the final approval of the version to be published.

## Funding

This study was supported by the Key Research and Development project of Shandong Province, China (Grant No. 2019GSF108230).

## Conflict of Interest

The authors declare that the research was conducted in the absence of any commercial or financial relationships that could be construed as a potential conflict of interest.

## Publisher's Note

All claims expressed in this article are solely those of the authors and do not necessarily represent those of their affiliated organizations, or those of the publisher, the editors and the reviewers. Any product that may be evaluated in this article, or claim that may be made by its manufacturer, is not guaranteed or endorsed by the publisher.
